# Human adipose-derived stem cells preconditioned with a novel herbal formulation Jing Shi attenuate doxorubicin-induced cardiac damage

**DOI:** 10.18632/aging.205026

**Published:** 2023-09-07

**Authors:** Dennis Jine-Yuan Hsieh, Bruce Chi-Kang Tsai, Parthasarathi Barik, Marthandam Asokan Shibu, Chia-Hua Kuo, Wei-Wen Kuo, Pi-Yu Lin, Cheng-Yen Shih, Shinn-Zong Lin, Tsung-Jung Ho, Chih-Yang Huang

**Affiliations:** 1 School of Medical Laboratory and Biotechnology, Chung Shan Medical University, Taichung, Taiwan; 2 Clinical Laboratory, Chung Shan Medical University Hospital, Taichung, Taiwan; 3 Cardiovascular and Mitochondrial Related Disease Research Center, Hualien Tzu Chi Hospital, Buddhist Tzu Chi Medical Foundation, Hualien, Taiwan; 4 Department of Biotechnology, Bharathiar University, Coimbatore, India; 5 Laboratory of Exercise Biochemistry, University of Taipei, Taipei, Taiwan; 6 Department of Kinesiology and Health Science, College of William and Mary, Williamsburg, USA; 7 Department of Biological Science and Technology, China Medical University, Taichung, Taiwan; 8 Ph.D. Program for Biotechnology Industry, China Medical University, Taichung, Taiwan; 9 Buddhist Compassion Relief Tzu Chi Foundation, Hualien, Taiwan; 10 Buddhist Tzu Chi Medical Foundation, Hualien, Taiwan; 11 Department of Neurosurgery, Hualien Tzu Chi Hospital, Hualien, Taiwan; 12 Integration Center of Traditional Chinese and Modern Medicine, Hualien Tzu Chi Hospital, Buddhist Tzu Chi Medical Foundation, Hualien, Taiwan; 13 Department of Chinese Medicine, Hualien Tzu Chi Hospital, Hualien, Taiwan; 14 School of Post-Baccalaureate Chinese Medicine, College of Medicine, Tzu Chi University, Hualien, Taiwan; 15 Department of Medical Research, China Medical University Hospital, China Medical University, Taichung, Taiwan; 16 Department of Medical Laboratory Science and Biotechnology, Asia University, Taichung, Taiwan; 17 Center of General Education, Buddhist Tzu Chi Medical Foundation, Tzu Chi University of Science and Technology, Hualien, Taiwan; 18 Graduate Institute of Basic Medical Science, China Medical University, Taichung City, Taiwan

**Keywords:** cardiac hypertrophy, reactive oxygen species, autophagy, chinese herbal medicine, adipose-derived stem cells

## Abstract

Pathological cardiac hypertrophy is a considerable contributor to global disease burden. Chinese herbal medicine (CHM) has been used to treat cardiovascular diseases since antiquity. Enhancing stem cell-mediated recovery through CHM represents a promising approach for protection against doxorubicin (Dox)-induced cardiac hypertrophy. Herein, we investigated whether human adipose-derived stem cells (hADSCs) preconditioned with novel herbal formulation Jing Si (JS) improved protective ability of stem cells against doxorubicin-induced cardiac damage. The effect of JS on hADSC viability and migration capacity was determined via MTT and migration assays, respectively. Co-culture of hADSC or JS-preconditioned hADSCs with H9c2 cells was analyzed with immunoblot, flow cytometry, TUNEL staining, LC3B staining, F-actin staining, and MitoSOX staining. The *in vivo* study was performed M-mode echocardiography after the treatment of JS and JS-preconditioned hADSCs by using Sprague Dawley (SD) rats. Our results indicated that JS at doses below 100 μg/mL had less cytotoxicity in hADSC and JS-preconditioned hADSCs exhibited better migration. Our results also revealed that DOX enhanced apoptosis, cardiac hypertrophy, and mitochondrial reactive oxygen species in DOX-challenged H9c2 cells, while H9c2 cells co-cultured with JS-preconditioned hADSCs alleviated these effects. It also enhanced the expression of autophagy marker LC3B, mTOR and CHIP in DOX-challenged H9c2 cells after co-culture with JS-preconditioned hADSCs. In Dox-challenged rats, the ejection fraction and fractional shortening improved in DOX-challenged SD rats exposed to JS-preconditioned hADSCs. Taken together, our data indicate that JS-preconditioned stem cells exhibit a cardioprotective capacity both *in vitro* and *in vivo*, highlighting the value of this therapeutic approach for regenerative therapy.

## INTRODUCTION

Epidemiological studies have shown that pathological cardiac hypertrophy is a major cause of morbidity and mortality worldwide. Pathological cardiac hypertrophy causes sudden heart failure, accounting for up to 17.3 million deaths annually [1]. In developed countries, hypertrophy-associated heart failure is typically associated with several risk factors, including stress, aging, diet, and physical inactivity [2, 3]. Chronic cardiac hypertrophy causes several cardiovascular conditions, including hypertension, ischemic disease, and heart failure [4]. Hypertrophy involves enhanced cardiac remodeling to increase left ventricular mass, causing left ventricular hypertrophy [5]. Doxorubicin (Dox), an effective anthracycline chemotherapeutic, is widely used in the treatment of several cancers, including lung, breast, prostate, and bone cancer, as well as leukemias [6]. However, various studies indicate that Dox can induce cardio toxicity inside and outside the cell by causing lipid peroxidation outside cardiomyocytes and free radical generation, organelle damage, and cellular signal imbalances inside cardiomyocytes, affecting heart function [6]. Furthermore, elevated Dox levels can cause cardiac hypertrophy leading to detrimental effects, such as cardiomegaly [7–9]. Typically, Dox causes excessive oxidative damage to the heart, promoting apoptosis [10]. Dox also has adverse effects on cytoplasmic calcium homeostasis [11]. Myocardial inflammation is driven by the activation of nuclear factor kappa B, a major transcription factor within the inflammatory response, and is also induced by Dox [12]. Transcription factor p53 is involved in upstream events leading to activation of the apoptotic pathway in mitochondria during Dox-induced cardiomyocyte death [13]. Developing novel therapies to reduce Dox cardiotoxicity is essential for improving its clinical efficacy.

Chinese herbal medicine is an effective and reliable treatment for several diseases [14, 15], and are widely used in various parts of the world. Importantly, CHM treatment is associated with few side effects [16, 17]. Owing to its high efficacy, few side effects, and low cost, CHM has been the focus of extensive research on cancer, cardiovascular diseases, diabetes, and coronavirus infectious disease (COVID-19), as well as stem cell therapy [18]. The novel herbal formulation Jing Si (JS) is used as tea in Taiwan. It contains various bioactive compounds and exhibits pharmacological properties that might protect cells under stress [19]. For instance, JS reduces DOX-related hypertrophic effects and DNA damage in H9c2 cells. It also enhances autophagic clearance in MPP-damaged SH-SY5Y neuroblasts. In addition, JS was shown to favorably regulate metabolism in a type II diabetes animal model. The growth of different cancer cell lines was suppressed by JS treatment. Further, JS was shown to promote stem cell homeostasis and offers cellular protection [19]. JS is composed of eight different CHM herbs and contains polyphenols, alkaloids, amino acids, organic acids, coumarins, vitamins, and phenols, which together act to exert beneficial effects on the human body.

Mesenchymal stem cell transplantation is an emerging approach in the field of regenerative medicine and influences growth factor secretion in cardiovascular disease [20, 21]. However, maintaining stemness alongside cardioprotective function is a major challenge after transplantation into the host. Adipose-derived stem cells (ADSCs) are an attractive option for stem cell therapy to regulate cardiac remodeling, as they are easily obtainable and have multi-lineage differentiation potential [22, 23]. ADSCs are able to regulate the “stem cell niche” in the host by stimulating the recruitment of endogenous stem cells to the transplant site and accelerating their differentiation. ADSCs may also act as free radical scavengers as well as a source of antioxidants and chaperone/heat shock proteins at sites of ischemia or injury [24]. This allows for detoxification of the microenvironment during stress conditions, which supports the remaining cells at these sites [24]. ADSCs also suppress the immune response and transfer healthy mitochondria to regulate aerobic metabolism. Compared to other stem cell types, ADSCs have notable advantages, such as their availability and low cost. They also secrete various growth factors, including hepatocellular growth factor (HGF), vascular endothelial growth factor (VEGF), insulin-like growth factor (IGF), and platelet-derived growth factor (PDGF), which confer cardioprotective effects under pathological conditions [25].

While Dox is an effective chemotherapeutic agent, its cardiotoxicity contributes to patient mortality. A limited number of studies have examined the effects of CHM on mesenchymal stem cell therapy for cardiac hypertrophy. While JS can promote stem cell homeostasis, there is no evidence to support whether it can enhance stem cell function. Therefore, we aimed to investigate whether JS could enhance the protective effects of ADSCs against Dox-induced cardiotoxicity *in vitro* and *in vivo*. We evaluated the effects of JS on human adipose-derived stem cells (hADSCs) via MTT and migration assays. Co-culture of hADSCs and H9c2 was followed by western blot, flow cytometry, TUNEL staining, immunoblot, F-actin staining, LC3B staining, and MitoSOX staining. For our *in vivo* study, Sprague-Dawley (SD) rats were subjected to M-mode echocardiography after receiving hADSCs. Our findings indicated that JS preconditioning improved the cardioprotective properties of stem cells against Dox, highlighting its value in regenerative therapy.

## MATERIALS AND METHODS

### Preparation and characterization of Jing Si herbal drink

The Jing Si herbal drink included 6 *g of* Ohwia caudate leaves*, 6 g of* Artemisia argyi leaves, *2 g of* Perilla frutescens leaves, *4 g of* Ophiopogon japonicas leaves, *4 g* of Platycodon grandifloras roots, *4 g* of Houttuynia cordata (Ophiopogonis Radix*) roots*, *2 g* of Glycyrrhiza uralensis (Glycyrrhizae radix*) roots, and 0.2 g of* Chrysanthemum × morifolium flowers. All herbs were bought from the local herbal store (Hualien, Taiwan) and finely powdered. The herbal mixture was added to 500 mL reverse osmosis water and concentrated to 50 mL via boiling. The preparation was spun down (slow speed) to remove the pellet and then filtered through a 0.45-μm filter [26].

### hADSCs and H9c2 cell culture

hADSCs were purchased from Thermo Fisher (Waltham, MA, USA) and cultured in mesenPRO RSTM basal medium supplemented with mesenPRO RSTM growth factor supplement (ThermoFisher) in an incubator at 37° C and 5% CO_2_. Cells were sub-cultured once the initial confluency reached 70%, with cells at passage 8 used for the experiments. H9c2 cells were obtained from American Type Culture Collection (USA) and cultured in Dulbecco’s Minimum Essential Medium (D5523, Sigma, Saint Louis, Missouri, USA) containing 10% fetal bovine serum (FBS) (Hyclone, Utah, USA) with 1% penicillin-streptomycin (Invitrogen Corp., California, USA), maintained at 37° C in a 5% CO_2_ incubator [27].

### Co-culture experiment

This method has been described in our previous report [28]. Briefly, hADSCs cultured in mesenPRO RSTM basal medium supplemented with mesenPRO RSTM growth factor supplement were seeded in the upper chamber of a hanging insert (Millipore, USA) and placed into the six-well culture plates containing H9c2 cells without contact to the lower chamber. H9c2 cells were cultured in six-well culture dishes. H9c2 cells cultured in Dulbecco’s Minimum Essential Medium containing 10% FBS with 1% penicillin-streptomycin were treated with Dox (1 μM), which was purchased from Sigma-Aldrich and diluted in dimethyl sulfoxide, for 24 h. After incubation for 24h, then cells were washed with PBS three times. The upper chamber with hADSCs preconditioned with JS was inserted into a 6-well dish for co-culture for 24 h. Finally, co-cultured H9c2 cells were washed with PBS three times and used for further experiments.

### MTT assay

The hADSCs cells were seeded at a density of 2 x10^5^ cells per well in 24-well plates. The cells were then treated with various concentrations (100–1,000 μg/mL) of JS for 24 h. MTT reagent (Sigma-Aldrich, MO, USA) was added at a concentration of 0.5 mg/mL for 4 h at 37° C. The medium was then discarded, and dimethyl sulfoxide was added for solubilization. Finally, the absorbance at 570 nm was measured using an automated microplate reader [29, 30].

### Western blot analysis

This method has been described in our previous reports [31–33]. Briefly, protein samples were extracted from H9c2 cells or heart tissues after treatment with lysis buffer (Tris-base [pH 7.4, 50 mM], EDTA [1 M], NaCl [0.5 M], beta-mercaptoethanol [1 mM], NP-40 [1%], IGEPAL CA-630 [Sigma-Aldrich], 10% glycerol, and protease inhibitor cocktail tablets [Roche, NY, USA]). Proteins were quantified, and an equal amount of protein from each sample was separated using sodium dodecyl sulphate–polyacrylamide gel electrophoresis. The proteins were then transferred onto polyvinylidene difluoride membranes (Millipore, Bedford, MA, USA), which were incubated with 5% blocking buffer for 1 h. The membranes were incubated with primary antibodies (mTOR [#2983] and p53 [#2524] from Cell Signaling [MA, USA], CHIP [sc-66830] and β-actin [sc-47778] from Santa Cruz Biotechnology [CA, USA]) at 4° C overnight. Finally, the membranes were incubated with secondary antibodies (horseradish peroxidase-conjugated anti-rabbit and mouse [Santa Cruz Biotechnology]) for 1 h at 25° C, and antibody binding was visualized using ECL western blotting luminal reagent (Santa Cruz Biotechnology) and the LAS-4000 mini (GE Healthcare Life Sciences) machine [34–36]. All chemicals were purchased from Sigma-Aldrich (St. Louis, MO, USA).

### F-actin, mitoSOX, and TUNEL staining

H9c2 cells were cultured in eight-well chamber slides (Greiner Bio-One, Monroe, North Carolina, USA). After reaching 70% confluence, the cells were fixed with 4% paraformaldehyde at room temperature for 1 h, washed thrice with PBS, and permeabilized with 0.1% Triton X-100 for 2 min. The cells were then incubated with rhodamine-phalloidin (Invitrogen), MitoSOX Red reagent (Invitrogen, Carlsbad, CA, USA), and TUNEL reagent (Roche Applied Science, Indianapolis, USA), according to the manufacturer’s protocol. After incubation, the cells were washed three times with PBS and counter-stained with DAPI (Abcam, Cambridge, UK) for 15 min for the nucleus staining. The whole field of vision was characterized using a fluorescence magnifying instrument (IX71, Olympus, Tokyo, Japan) associated with an imaging framework (DP2-BSW, Olympus). The quantification results were further assessed and plotted using GraphPad Prism software.

### LC3B staining

H9c2 cells were cultured in eight-well chamber slides (Greiner Bio-One, Monroe, North Carolina, USA). After reaching 70% confluence in DMEM containing 10% FBS, the cells were fixed with 4% paraformaldehyde in 1× PBS for 1 h at room temperature. Permeabilization solution (0.5 mL, 0.1% Triton X-100 in 0.1% sodium citrate) was added to each well on ice for 2 min without shaking. Blocking buffer (2% BSA) was added in each well to avoid non-specific binding. Primary antibody against LC3B (#2775, Cell Signaling Technology, 1:100, 500 μL) was added to each well and incubated at 4° C for 12 h. Subsequently, diluted fluorescent secondary antibody Alexa Fluor^®^ 488 goat anti-rabbit IgG (A11008, Invitrogen, 1:100, 500 μL) was added to each well and incubated at 25° C for 1 h. DAPI (500 μL, 10000× diluted) was added to each well. The plates were incubated for 30 min at 25° C, in the dark. Finally, after washing with PBS, the cells were observed under fluorescence microscope (IX71, Olympus, Tokyo, Japan).

### Animal experiments

The animals were purchased from BioLASCO Taiwan Co., Ltd. (Taipei, Taiwan). Eight-week-old SD rats were maintained under a 12-h light/dark cycle at 55 ± 10% humidity and 22 ± 2° C, with access to food and water. Healthy SD rats were allocated into five groups (n = 4 per group) and treated once every 2 weeks for a total 4 weeks, as follows: (Group I) SD rats (control), (Group II) SD rats treated with Dox (7.5 mg/kg) for 4 consecutive weeks to achieve a total concentration of 30 mg/kg, (Group III) SD rats treated with Dox after oral administration of JS (300 mg/kg), (Group IV) SD rats treated with Dox and JS (50 μg/mL)-preconditioned hADSCs (1×10^6^ cells/rat via tail vein injection), and (Group V) SD rats treated with Dox and JS (100 μg/mL)-preconditioned hADSCs (1×10^6^ cells/rat via tail vein injection). After treatment, heart function was analyzed using M-mode echocardiography before the rats were euthanized. Left ventricular internal end-diastolic dimensions (LVIDd), left ventricular internal end-systolic dimensions (LVIDs), stroke volume (SV), and end diastolic volume (EDV) were examined via echocardiography. Fractional shortening (FS) was determined as per the following formula: FS (%) = [(LVIDd –LVIDs)/ LVIDd] × 100. The e*jection fraction* (EF) was determined as: EF (%)=SV/EDV × 100 [37–39]. Thereafter, all animals were euthanized via CO_2_ asphyxiation. All hearts were collected and stored at -80° C for further experiments.

### Analysis of apoptosis by flow cytometry

Flow cytometry analysis was performed using a double staining Annexin V-FITC and propidium iodide (PI) apoptosis detection kit (BD Biosciences, USA), according to the manufacturer’s protocol for *in vitro* analysis. After processing using the kit, apoptosis analysis was carried out using a FACS CantoTM system (BD Biosciences, USA) at the FACS Core Facility, Tzu-chi Hospital Research Center, Taiwan. The apoptotic cells were gated (n=10,000 cells), and the proportion of apoptotic cells was calculated by adding the numbers of cells in the Q2 (late apoptosis) and Q4 (early apoptosis) quadrants.

### Migration assay

A migration assay was performed as previously described [40]. In brief, 2×10^5^ cells per well were seeded into the chambers of Transwell plates in serum-free media, and the lower chamber was filled with 10% FBS as an attractant. The plates were incubated for 24 h at 37° C with 5% CO_2_. After treatment, the chamber membrane was treated with 4% paraformaldehyde to fix the cells and stained with crystal violet. Cells that migrated to the lower chamber were observed using an OLYMPUS^®^ BX53 microscope (Tokyo, Japan).

### Statistical analysis

All data are expressed as the mean ± standard error of the mean (SEM). Quantifications performed in triplicate were analyzed using one-way ANOVA in Prism GraphPad 5 software. P-values lower than *p <0.05, **p <0.01, and ***p <0.001 were considered statistically significant.

### Availability of data and material

The raw data used and/or analyzed during the current study are available from the corresponding author on reasonable request. The authors confirm that the data supporting the findings of this study are available within the article.

### Consent for publication

The authors agree the publication.

## RESULTS

### JS-preconditioned hADSCs exerted cytoprotective effects on Dox-challenged H9c2 cells

**Figure 1:**
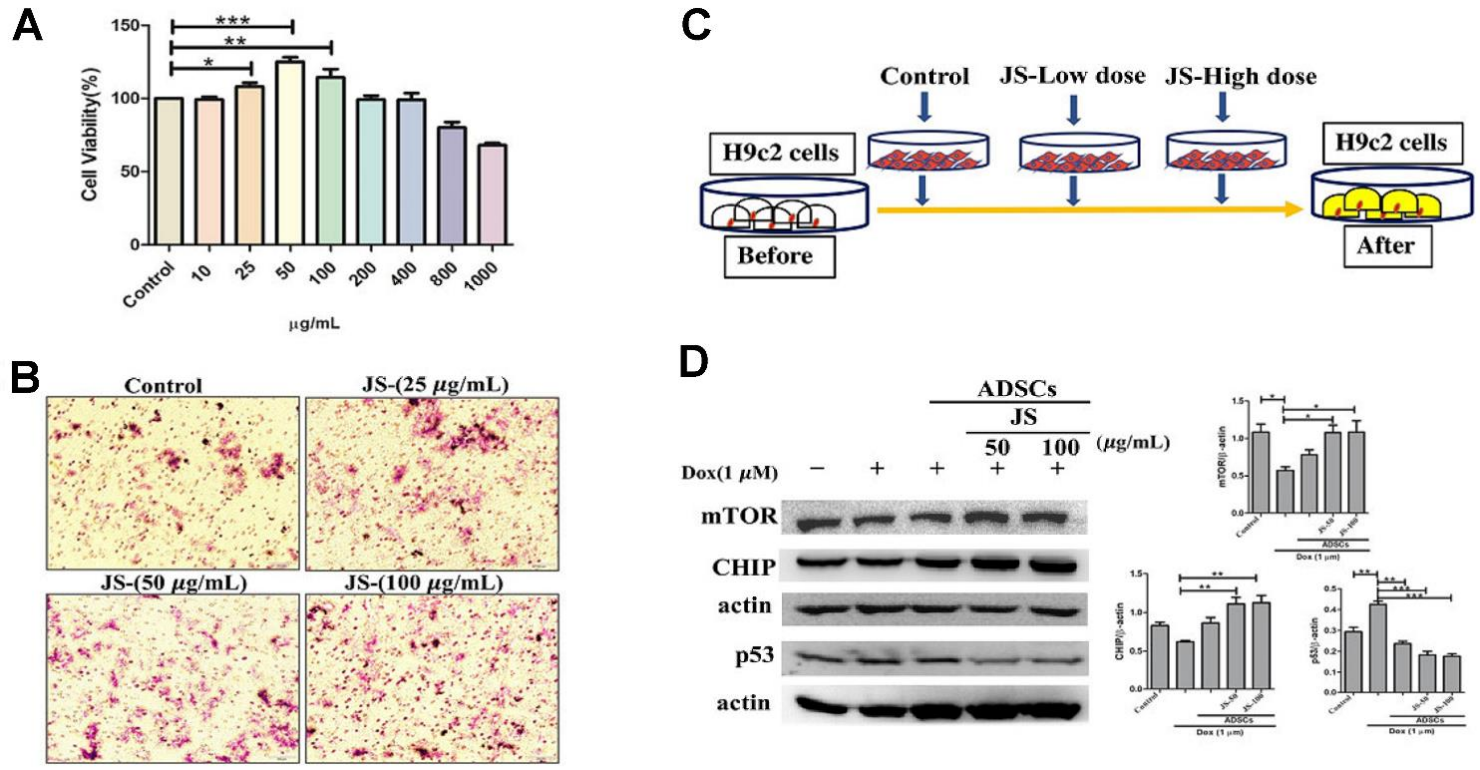
**Jing Shi-preconditioned human adipose-derived stem cells (hADSCs) enhanced cytoprotective effects of doxorubicin-challenged H9c2 cells.** (**A**) Cell viability assay indicating cell viability of human adipose-derived stem cells (hADSCs) treated with Jing Shi. (**B**) Transwell migration assay showing that Jing Shi-preconditioned hADSCs showed more migration efficiency (pink color) compared with that of the control. (**C**) Schematic diagram outlining the strategy for co-culturing hADSC and doxorubicin-challenged H9c2 cells. (**D**) Immunoblot results showing that Jing Shi-preconditioned hADSCs co-cultured with doxorubicin-challenged H9c2 cells increased mTOR and CHIP expression and attenuated apoptosis marker p53 protein expression in H9c2 cells. Experiments were performed in triplicate*.* Data are presented as means ± SEM. *p <0.05, **p <0.01, and ***p <0.001 were considered significant.

**Figure 2:**
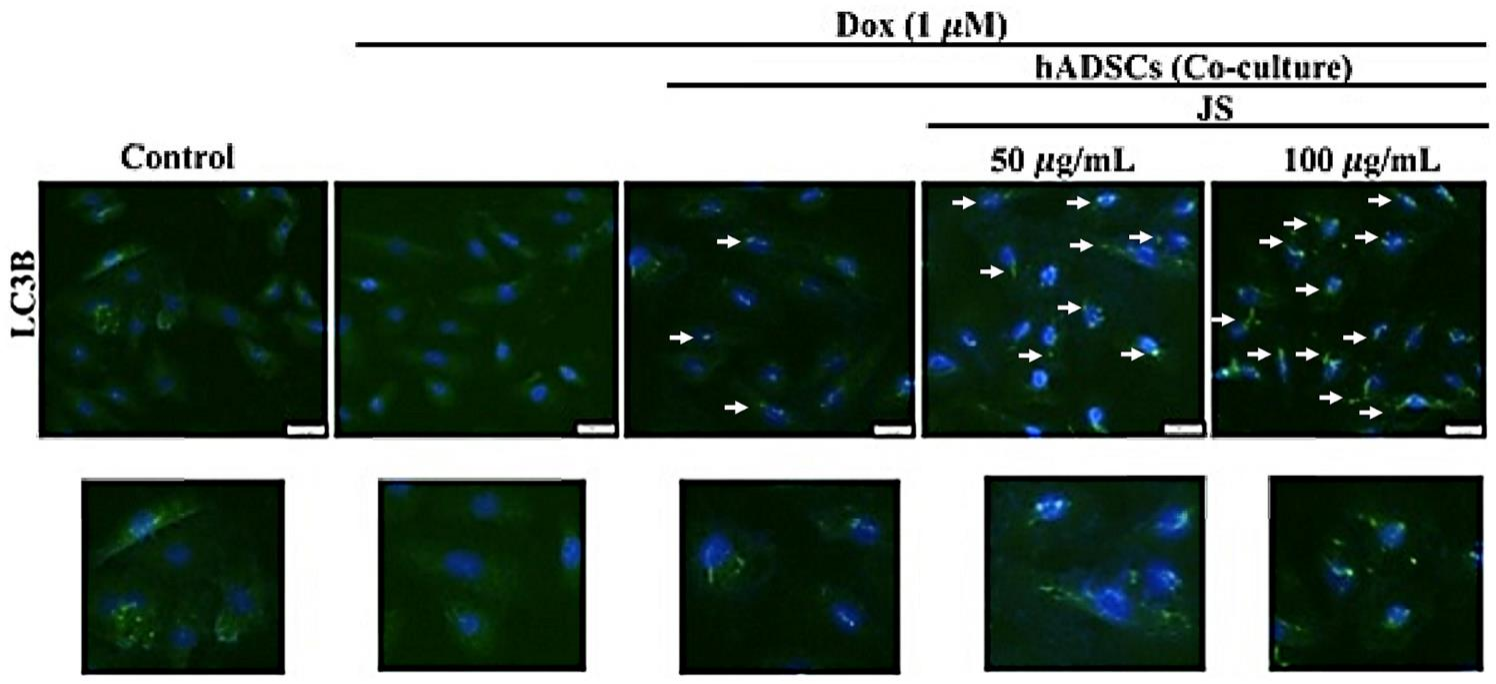
**Jing Shi-preconditioned human adipose-derived stem cells (hADSCs) enhanced autophagy of doxorubicin-challenged H9c2 cells.** The essential autophagic marker LC3B expression in Dox-challenged H9c2 cells was also exhibited lower expression. However, co-culture with hADSCs preconditioned with 50 or 100 μg/mL JS significantly induced the upregulated expression of LC3B in H9c2 cells. Scale bar was 100 μm.

To evaluate the effect of JS on hADSC viability, we performed an MTT assay. The results indicated that after 24 h of treatment, hADSC viability increased under low doses of JS (25, 50, 100 μg/mL), whereas high doses (up to 800 μg/mL) exhibited low cytotoxicity (Figure 1A). Through transwell migration transwell migration assay to examine the effect of JS treatment on migration efficiency, we observed that hADSC migration increased in a dose-dependent manner at low dose concentrations of JS (Figure 1B). We consider JS contains various bioactive compounds that may regulate the microenvironment by stimulating cells to secrete soluble trophic factors that regulate stemness, through autocrine and paracrine mechanisms. To determine whether JS-preconditioned hADSCs exert a paracrine effect on Dox-challenged H9c2 cells, we performed a co-culture experiment, summarized in a schematic diagram (Figure 1C). The data from western blot analysis revealed that Dox-challenged H9c2 cells exhibited a decreased expression of mammalian target of rapamycin (mTOR) and carboxy terminus Hsp70-interacting protein (CHIP) protein, both of which regulate autophagic flux. Meanwhile, co-culture with hADSCs preconditioned with 50 or 100 μg/mL JS significantly upregulated mTOR and CHIP in H9c2 cells, while downregulating apoptosis marker p53 in a dose-dependent manner (Figure 1D). Similarly, the autophagic marker LC3B expression in Dox-challenged H9c2 cells was also exhibited lower expression. However, co-culture with hADSCs preconditioned with 50 or 100 μg/mL JS significantly induced the upregulated expression and aggregation of LC3B in Dox-challenged H9c2 cells (Figure 2). Because cells utilized autophagy to eliminates protein aggregates and damaged organelles, and by promoting bioenergetic homeostasis [41], these data suggest that JS-preconditioned hADSCs exert a cytoprotective effect to maintain a healthy cellular environment in Dox-challenged H9c2 cells through the stimulation of autophagic mechanism.

### JS-preconditioned hADSCs inhibited the apoptosis of Dox-challenged H9c2 cells

**Figure 3:**
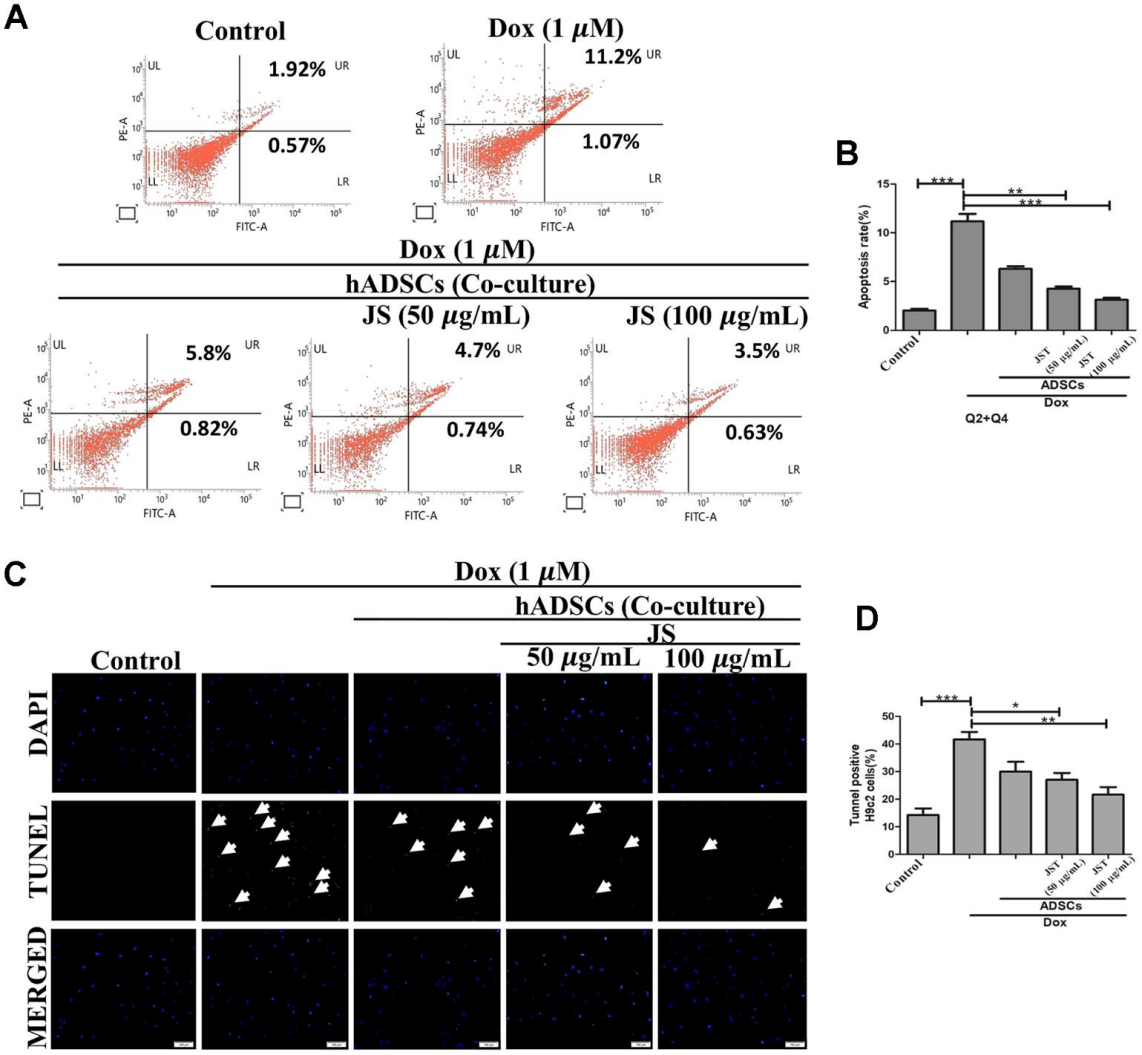
**Jing Shi-preconditioned human adipose-derived stem cells (hADSCs) co-cultured with doxorubicin-challenged H9c2 cells decreased doxorubicin-induced apoptosis.** (**A**, **B**) Flow cytometry analyzing cell apoptosis in H9c2 cells after doxorubicin induction with different JS-preconditioned hADSCs treatment groups versus control. Jing Shi-preconditioned human adipose-derived stem cells (hADSCs) remarkably decreased cell apoptosis in doxorubicin-challenged H9c2 cells (**C**, **D**) TUNEL assay indicating apoptotic cells (green color fluorescence) in control and different treatment groups. DAPI counter stain indicates the nucleus. The number of TUNEL positive cells decreased when doxorubicin-challenged H9c2 cells were co-cultured with Jing Shi-preconditioned hADSCs. Experiments were performed in triplicate. Data are presented as means ± SEM. *p <0.05, **p <0.01, and ***p <0.001 were significant.

Several studies have reported that exposure to Dox promotes apoptosis in H9c2 cells [42, 43]. To examine whether JS-preconditioned hADSCs protects against Dox-induced apoptosis, we performed flow cytometry analyses of co-cultured H9c2 cells after Annexin V staining. After co-culture of JS-preconditioned hADSCs with H9c2 cells that were treated with Dox for 24 h, we determined the total proportion of apoptotic H9c2 cells by quantifying the number of cells undergoing late apoptosis (upper right quadrant; Q2) and early apoptosis (lower right quadrant; Q4). The apoptotic population was significantly reduced in a dose-dependent manner in Dox-challenged H9c2 cells co-cultured with JS-preconditioned hADSCs, as compared to that following co-culture with untreated ADSCs (Figure 3A, 3B). These data suggest that JS conditioning enhanced stem cell viability, thus maintaining a cytoprotective microenvironment, which helps nullify the cytotoxic effects of Dox exposure in H9c2 cells.

In concordance with the flow cytometry results, we also observed via TUNEL assay that Dox causes apoptosis. Results from TUNEL analyses indicated that the Dox challenge significantly upregulated the number of apoptotic cells, whereas co-culture with JS-preconditioned hADSCs reduced the number of apoptotic cells in a dose-dependent manner (Figure 3C, 3D). Altogether, these results confirm that the JS-preconditioned hADSCs act as a booster to maintain the health of H9c2 cells in response to Dox.

### JS-preconditioned hADSCs inhibited Dox-induced cellular hypertrophy and mitochondrial ROS generation in H9c2 cells

**Figure 4:**
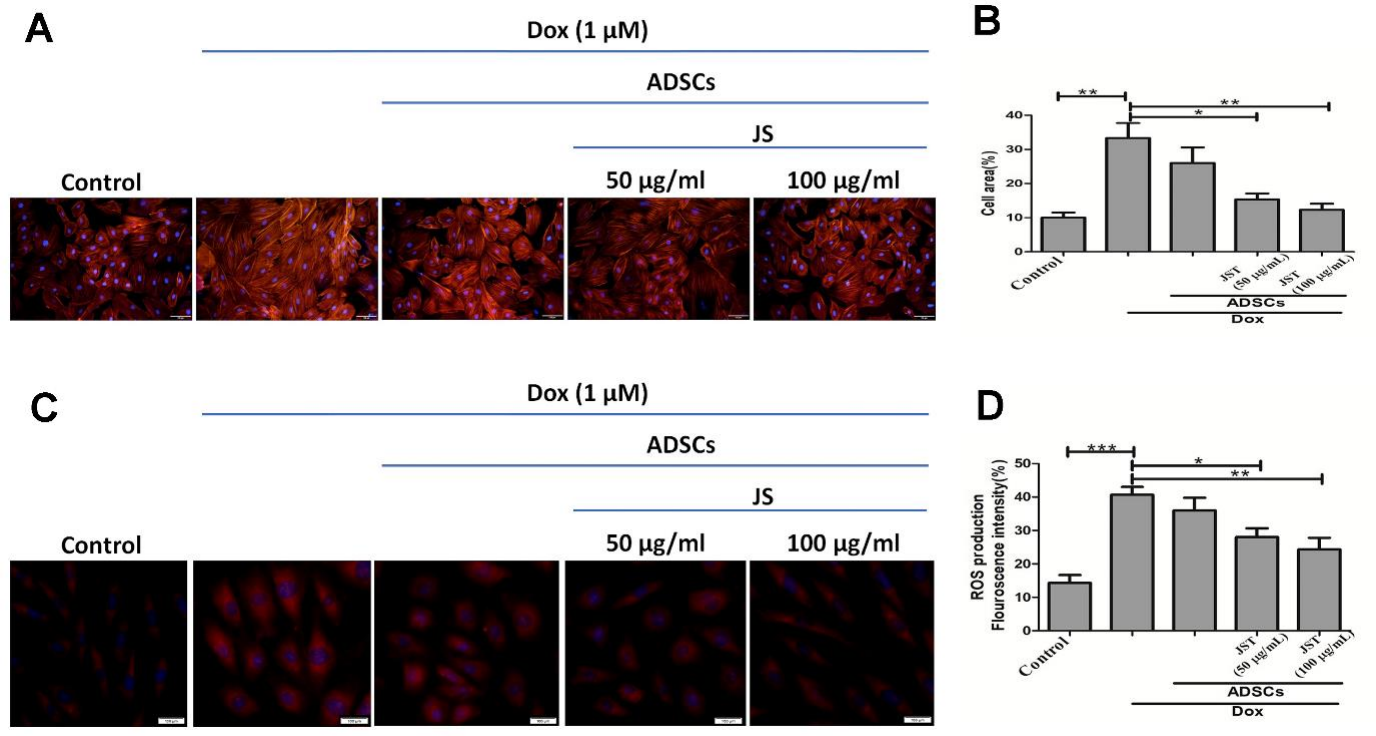
**Doxorubicin-challenged H9c2 cells co-cultured with Jing Shi-preconditioned human adipose-derived stem cells (hADSCs) presented less hypertrophy and low-level mitochondrial reactive oxygen species.** (**A**) F-actin staining detecting hypertrophy with or without Doxorubicin induction in H9c2 cells after co-culture with human adipose-derived stem cells (hADSCs). (**B**) quantitative analysis of cell area for Doxorubicin-challenged H9c2 cells. Jing Shi-preconditioned hADSCs significantly reduced hypertrophy in doxorubicin-challenged H9c2 cells (**C**, **D**) MitoSOX staining detecting mitochondrial reactive oxygen species and their quantitative analysis. Doxorubicin-challenged H9c2 cells showed the least mitochondrial reactive oxygen species levels after co-culture with Jing Shi-preconditioned hADSCs. Experiments were performed in triplicate. Data are presented as means ± SEM. *p <0.05, **p <0.01, and ***p <0.001 were significant.

Dox has been shown to induce hypertrophy in H9c2 cells [44]. Further, prolonged exposure to Dox is associated with reduced heart function, which can lead to heart failure and sudden cardiac arrest. To validate the hypertrophic response to Dox in H9c2 cells, we performed F-actin staining, the results of which indicated that Dox challenge increased H9c2 cell size, whereas co-culture with hADSCs or JS-preconditioned hADSCs reduced the hypertrophic effects of Dox and normalized the cell size of H9c2 cells. Quantitative analysis confirmed that there was a significant increase in H9c2 cells after incubation in Dox for 24 h, but JS-hADSC co-culture significantly had better effect in Dox-treated H9c2 cells and the effect was in a dose-dependent manner (Figure 4A, 4B). Taken together, these findings demonstrated that co-culture with JS-preconditioned hADSCs nullified the hypertrophic response in Dox-treated H9c2 cells.

Dox induces oxidative stress through the generation of reactive oxygen species (ROS) in H9c2 cells [45]. Further, ROS are primarily responsible for the cellular damage and apoptosis associated with Dox exposure. An earlier study showed that increased mitochondrial ROS regulates cardiotoxicity in H9c2 cells [46]. We therefore next to analyze the mitochondrial superoxide generation in JS-treated H9c2 cells challenged with Dox, using MitoSOX red staining. The fluorescence results indicated that Dox enhanced ROS generation, whereas JS-preconditioned hADSCs nullified the increase in ROS in a dose-dependent manner in H9c2 cells (Figure 4C, 4D). Taken together, our data suggest that JS-preconditioned hADSCs exert a neutralizing effect on ROS generated in response to Dox challenge in H9c2 cells.

### JS and JS-preconditioned hADSCs regulated cardiac function in Dox-challenged SD rats

**Figure 5:**
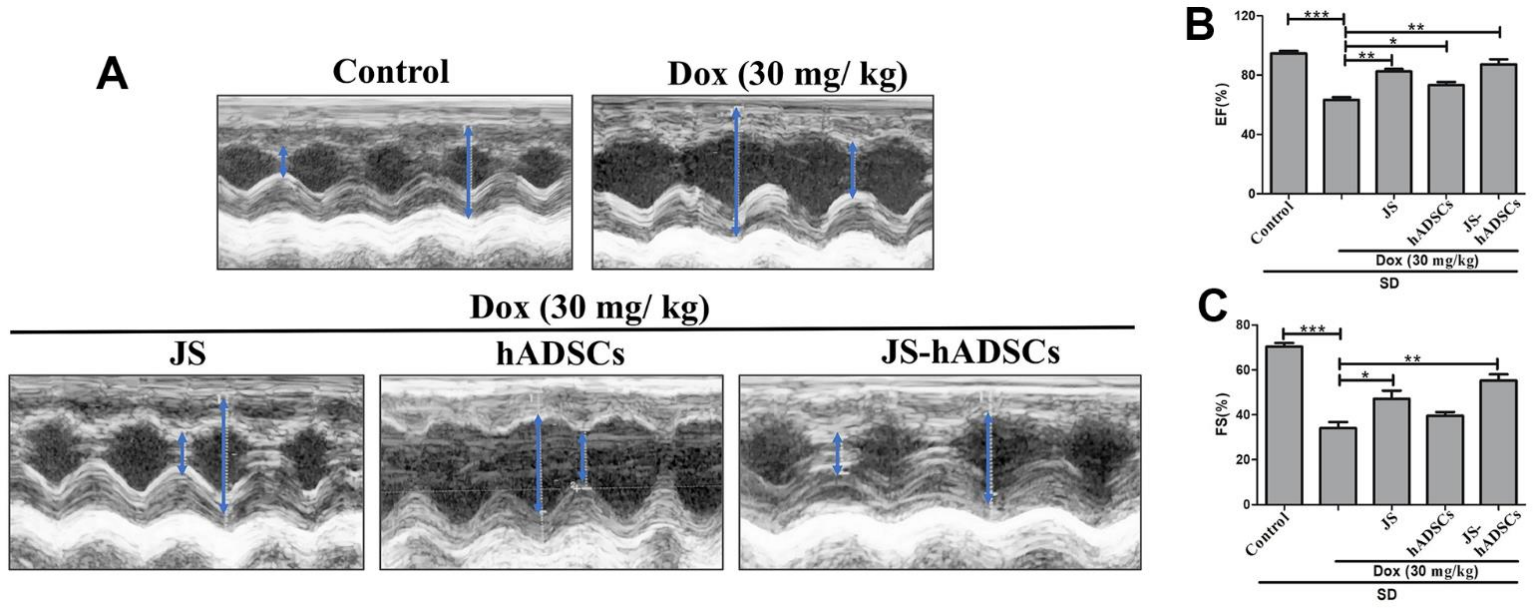
**Role of Jing Shi and Jing Shi-preconditioned human adipose-derived stem cells (hADSCs) on cardiac function in doxorubicin-challenged Sprague–Dawley rats.** (**A**) M-mode echocardiography results showing contractility functions (i.e., left ventricular internal diameter end diastole and end systole. (LVIDd and LVIDs)) of all rat groups, indicated by the blue arrow. Doxorubicin-challenged Sprague–Dawley rats treated with Jing Shi-preconditioned human adipose-derived stem cells (hADSCs) showed similar patterns to that of the control group. (**B**, **C**) The ejection fraction (EF%) and fractional shortening (FS%) of control, doxorubicin, and various treatment groups. Jing Shi-preconditioned hADSCs showed an improved repair of heart function in Doxorubicin-challenged Sprague–Dawley rats*.* Experiments were performed in triplicate. Data are presented as means are represented as means ± SEM. *p <0.05, **p <0.01, and ***p <0.001 were significant.

The results from our *in vitro* analysis showed that low dose JS enhanced the survival and migration of hADSC. Furthermore, JS-preconditioned hADSCs provide a suitable microenvironment that supports H9c2 cells in nullifying the detrimental effects of Dox. To validate these findings in an *in vivo* model, we next investigated whether JS-preconditioned hADSCs provide cardioprotective effects in Dox-challenged SD rats. Interestingly, we found that both JS and JS-preconditioned hADSCs provided cardioprotective effects to rats challenged with Dox, compared to the only Dox-treated group. The left ventricular internal diameter end diastole (LVIDd) and end systole (LVIDs) values of the JS groups showed a remarkable contractility function after the treatment, with the JS-preconditioned hADSCs exhibited a more pronounced favorable effect (Figure 5A). Similarly, the ejection fraction (EF%) and fractional shortening (FS%) also indicated a significant improvement in cardiac function following JS and JS-hADSC treatment (Figure 5B, 5C). Along with our *in vitro* results, these findings suggest that JS-preconditioned hADSCs could regulate mitochondrial ROS and suppress apoptosis to maintain cardiac function following Dox challenge *in vivo*.

## DISCUSSION

We have recently demonstrated that resveratrol-preconditioned ADSCs increases the regenerative capacity of diabetic hearts via the Sirt1/Akt signaling pathway [47]. Furthermore, *Alpinia oxyphylla* extract-preconditioned ADSCs attenuate mitochondria-mediated cardiac apoptosis and maintain cardiac function in an aging rat model [48]. Hence, in the current study, we aimed to evaluate the cardioprotective effects of JS-preconditioned hADSCs against Dox-induced cardiac damage and found that JS-preconditioned hADSCs attenuated Dox-induced cardiac damage *in vitro* and *in vivo*.

Autophagy is an important phenomenon that maintains the homeostasis mechanism of the cells during stress conditions. Maintaining autophagic flux via the CHIP and mTOR proteins is an important cellular approach to mimic the experiment with Dox challenge [49]. Previous studies have indicated that Dox challenge causes ROS generation, which leads to cardiac apoptosis via p53 upregulation [44]. Another study also emphasized that Dox attenuated autophagy and co-chaperone activity in SD rats after treatment [50, 51]. Autophagy is an important quality control mechanism in healthy cells, and its cytoprotective effects involve the removal of unfolded and damaged proteins [52]. Our results identified similar mechanisms, with our *in vitro* data revealed that JS-hADSC treatment against Dox challenge regulated expression of the autophagy marker mTOR and the co-chaperone CHIP, in addition to downregulating the apoptosis marker p53. A previous report shows that CHIP E3 ligase regulates p53 degradation [53] which is in concordance with our western blot analysis. JS may regulate this by its bioactive compounds that leads to maintenance of the mesenchymal stem cells microenvironment, enabling these cells to regulate the secretion of soluble trophic factors and to regulate autophagy in Dox-challenged H9c2 cells. Similarly, JS-hADSC treatment may control mitophagy to reduce apoptosis in Dox-challenged H9c2 cells. For example, Luteolin, a natural compound in vegetables and fruits, activates mitochondrial autophagy to attenuate Dox-induced cardio toxicity in cardiomyocytes [54]. In the present study, mTOR and CHIP expression increased, but p53 expression decreased after co-culture with JS-hADSC. Previous literature mentions that mTOR inhibition immediately changes mitochondrial function [55]. Mitochondrial autophagy is also regulated by CHIP expression and localization [56]. Thus, p53 inhibits Parkin-mediated mitochondrial autophagy resulting in mitochondrial dysfunction [57]. Besides, LC3B is the extensively accepted marker for autophagy activity assessment as it is important for the autophagy mechanism [58]. Hence, we consider that JS-hADSC treatment can regulate autophagy to maintain a healthy cellular environment in Dox-challenged H9c2 cells.

Growth factor secretion by mesenchymal stem cells regulates various signaling pathways, such as the IGF1-IGF1R-AKT-mTOR pathway [59]. Our *in vitro* studies provided strong evidence that JS enhanced the migratory ability of hADSCs after treatment in a dose-dependent manner, which indicates the migration efficiency of the stem cells after transplantation. The damaged myocardial tissue secretes SDF1a, which helps to recruit stem cells via the chemokine marker CXCR4 to repair vascular damage [60, 61]. Several studies have focused on the paracrine activity of mesenchymal stem cells to mitigate vascular damage after stress [62–64]. ADSCs have the potential to secrete several growth factors, anti-inflammatory cytokines, and chemokines that mediate cardiac injury repair. These secreted soluble trophic factors promote migration, cell proliferation, and cytoprotection. Under pathological conditions, stem cells provide a supportive microenvironment by producing antioxidant and antiapoptotic factors to nourish the damaged cells [65]. Mesenchymal stem cells also secrete anti-fibrotic and angiogenic factors that modulate protection of the heart [66]. These pleotropic growth factors, such as VEGF, HGF, IGF, and PDGF, enhance cardiac repair during chronic stress conditions (e.g., pathological hypertrophy). Various preconditioning mechanisms have been applied to enhance the ability of these growth factors to restore blood flow during damaged conditions such as myocardial infarction and pathological hypertension. Here, we used JS-preconditioned hADSCs, and examined their cardioprotective capabilities in both *in vitro* and *in vivo* contexts. The paracrine effects of JS-preconditioned hADSCs involve promoting a healthy microenvironment that protect H9c2 cells against the stress mediator doxorubicin. Based on our results, we hypothesized that ADSCs treated with JS act in a paracrine manner to exert a cardioprotective effect against Dox-induced cardiac damage that leads to enhanced EF (%) and FS (%) functions.

## CONCLUSIONS

**Figure 6:**
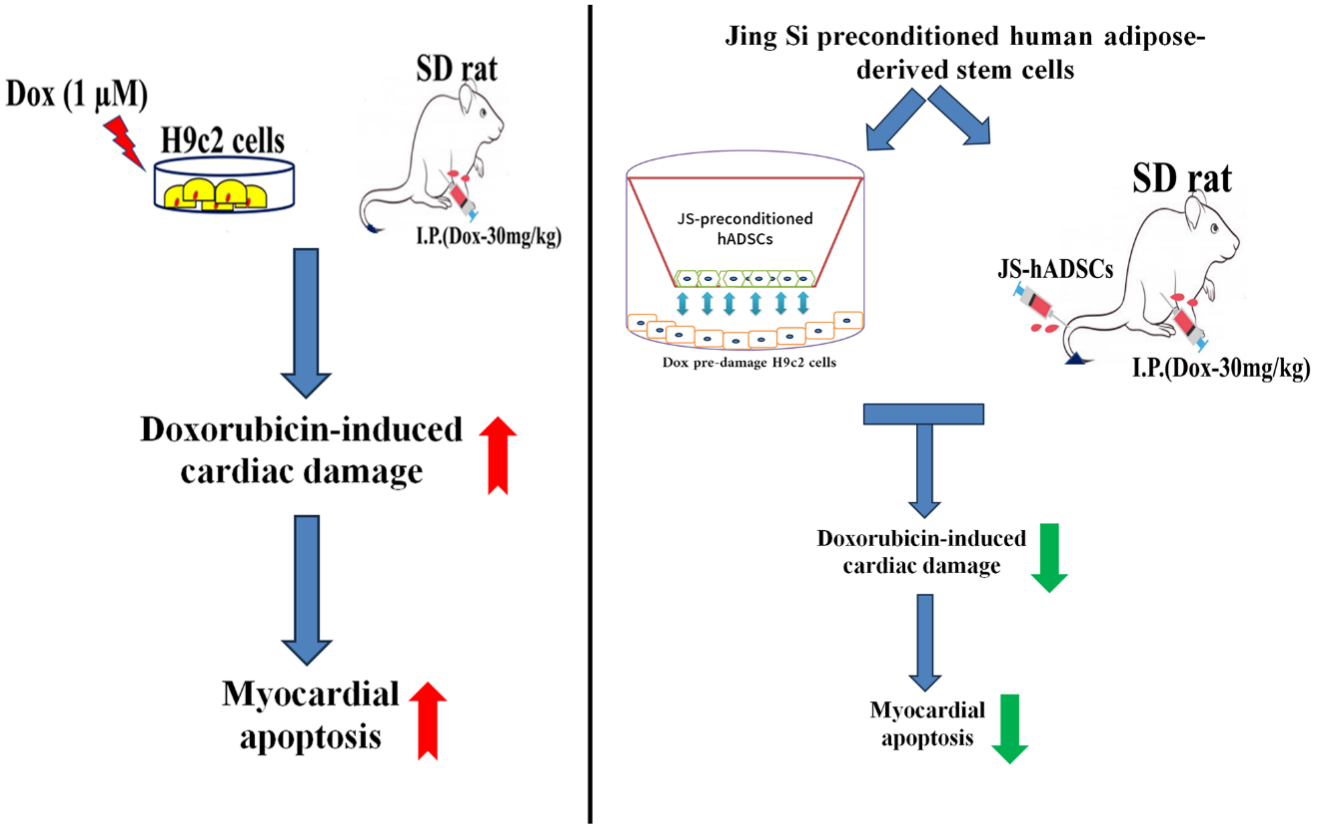
Graphical representation of the cardioprotective effects of Jing Shi and Jing Shi-preconditioned human adipose-derived stem cells (hADSCs) against Doxorubicin (Dox) induction in *in vitro* and *in vivo* models.

In this study, we reported that JS-preconditioned hADSCs have exhibited protective effects in dox-induced hypertrophic conditions in both *in vitro* and *in vivo* conditions (Figure 6). The *in vitro* model demonstrated that JS-preconditioned hADSCs has cardioprotective effects by regulating mitochondrial ROS, cardiac hypertrophy, and apoptosis in Dox-challenged H9c2 cells via activation of autophagy. Our *in vivo* data suggest that the preconditioning of hADSCs enhance cardiac function that might be regulated by secreting growth factors and regulating cell viability, as well as improving migration efficiency. Taken together, our data indicate that JS preconditioning of hADSCs augments their cardioprotective effects in reducing ROS and apoptosis in H9c2 cells. To maintain the viability after transplantation is a greater challenge in stem cell therapy. So, we presume that this therapeutic strategy can also enhance cardiac function by enhancing the viability and migratory ability of the cells against Dox damage conditions. Our study shows that JS-preconditioned stem cells regulate the cardioprotective mechanism, both *in vitro* and *in vivo*, and these results suggest that this therapeutic approach is important for further investigation as a regenerative therapy.
